# Insights into Heavy Metals Leakage in Chelator-Induced Phytoextraction of Pb- and Tl-Contaminated Soil

**DOI:** 10.3390/ijerph16081328

**Published:** 2019-04-12

**Authors:** Xuexia Huang, Dinggui Luo, Xiangxin Chen, Lezhang Wei, Yu Liu, Qihang Wu, Tangfu Xiao, Xiaotao Mai, Guowei Liu, Lirong Liu

**Affiliations:** 1 School of Environmental Science and Engineering, Guangzhou University, Guangzhou 510006, China; huangxuexia66@163.com (X.H.); chanxx0727@163.com (X.C.); wlz2016@gzhu.edu.cn (L.W.); liuyu@gzhu.edu.cn (Y.L.); tfxiao@gzhu.edu.cn (T.X.); maixiaotao@e.gzhu.edu.cn (X.M.); liuguowei@e.gzhu.edu.cn (G.L.); leonliutiming@e.gzhu.edu.cn (L.L.); 2 Linköping University—Guangzhou University Research Center on Urban Sustainable Development, Guangzhou University, Guangzhou 510006, China; 3 Guangdong Provincial Key Laboratory of Radionuclides Pollution Control and Resources, Guangzhou University, Guangzhou 510006, China; wuqihang@gzhu.edu.cn; 4 Key Laboratory for Water Quality and Conservation of the Pearl River Delta, Ministry of Education, Guangzhou University, Guangzhou 510006, China

**Keywords:** phytoextraction, thallium, lead, maize, DTPA, oxalic acid, leaching

## Abstract

Chelators including DTPA (diethylene triamine pentaacetic acid) and oxalic acid were selected for inducing phytoextraction of heavy metals (HMs) from Pb-, Tl-, and Pb-Tl- contaminated soil, in which heavy metals leakage was highly remarkable. Results showed that compared with the control group without chelating agent under planting conditions, the extraction efficiency (i.e., uptake coefficient) of Pb, Tl increased by 86%, 43% from Pb-Tl- contaminated soil in the presence of oxalic acid, and there was no significant change in heavy metal leakage under rainfall conditions. It was the best phytoremediation scheme in this work. Under rainfall conditions, the HMs concentration in the leachate showed a linear decreasing trend. Acid rain promoted the leakage of heavy metals, and the average leached amount of Tl increased by 1.47 times under acid rain conditions. However, for Pb, DTPA was the main influencing factor, followed by acid rain.

## 1. Introduction

The excessive release of heavy metals (HMs) from industries results in soil contamination, which is a serious threat to the ecological environment and human health [1,2], and is becoming a global environmental problem. Methods of soil remediation are highly concerned. Among all the remediation methods, phytoextraction, which uses plants to extract HMs from soil, has been widely applied due to its economic and environmental benefits [3,4]. 

Hyperaccumulators and tolerant plants have been extensively concerned in phytoextraction processes [5]. Though hyperaccumulators show good performance in HMs extraction and transference, their applications are often limited by their low biomass and poor environmental adaptability [6,7,8]. For tolerant plants, although the unit efficiency of absorbing heavy metals is low, due to the high biomass, the total amount of HM absorption can be comparable to that of hyperaccumulators. 

Typical tolerant plants including *Zea mays*, sunflower [9], spinach [10], cabbage, and chrysanthemum, etc., [11,12,13,14,15] are widely considered in phytoextraction processes. Among them, *Z. mays* is a promising species for HMs phytoextraction from contaminated soil [16]. It is reported that it has a high biomass and good characteristics of heavy metal (e.g., Pb and Cd) enrichment and transfer [17]. More importantly, HMs are mainly found in the roots, stems, and leaves. HMs are found in low concentrations in grain [18]. It has been noted that the stalks of maize can be used as energy materials for fuel production (e.g., ethanol and biogas) [19]. Therefore, phytoextraction by *Z. mays* has many advantages including environmental protection, grain production, and energy production as-above mentioned.

Unfortunately, most of the HMs in the soil have low bioavailability, which is not conducive to plant extraction. Therefore, chelators are often added to increase the absorption efficiency of HMs by tolerant plants since they can activate HMs from the surfaces of soil particles into soil solutions by forming soluble complexes with heavy metals [4,20,21]. DTPA and oxalic acid are two commonly used artificial chelating agents and small molecular organic acids, respectively, which have significant effects on the extraction of heavy metals from plants [22,23], especially DTPA. For example, the Pb concentration in the upper parts of maize treated by DTPA was 9.5 times higher than that of the control sample [24], the Pb concentration in *L. perenne* was 2.09-fold higher than that of the control sample [25], and the bioconcentration factors of Pb in *Eucalyptus camaldulensis* Dehnh increased by 86%. In addition, with the addition of DTPA, the accumulation of Tl in barley was enhanced markedly [26]. Salix taxa performs at a higher extraction efficiency of heavy metals from Cu, Pb, and Zn contaminated soil, which is mainly related to the rhizosphere secreting more small molecular organic acids, such as oxalic acid and malic acid [27]. The bioavailability of heavy metals reached the maximum at the second day after the addition of the chelating agent, EDTA (ethylene diamine tetraacetic acid), and its combination with small molecular organic acids (citric acid and oxalic acid) could significantly improve the extraction efficiencies of Cd and Zn. Their concentrations in the shoots and roots of potherb mustard were significantly amplified by 1.7, 2.15 and 1.93, 2.7 folds than those of control samples, respectively [28].

Although the chelating agents induced the extraction efficiencies of heavy metals from contaminated soil, it also may lead to the leakage of heavy metals under rainfall conditions and even result in the risk of groundwater pollution [29]. However, the study on the HMs leakage during phytoextraction is very limited at present. Studies only focused on theoretical inference, leaching experimental research under non-planting conditions and non-acid rain conditions [30,31,32,33]. For example, Ning et al. [34] suggested that all the heavy metals in the soil were activated by chelating agents, but only some of them were absorbed by plants. So, it is theoretically inferred that there is a risk of multiple HMs leaching. The leaching experiments under non-planting conditions showed that the addition of EDTA would significantly increase the concentration of heavy metals in the leachate, especially during the periods of high rainfall [29]. There is a good correlation between plant extraction and leaching of HMs (Cu, Cd, Pb, Zn, Ni), with the exception of Cr [35]. However, few studies focused on the HMs leaching under acid rain conditions. Moreover, little attention was paid to Tl leaching.

Guangdong province is rich in metal mineral resources. Tl is a dispersive associated element [36]. Due to the exploitation of mineral resources, the coexistence of Pb, Tl, and other heavy metals has become a typical type of soil pollution [37,38,39,40]. In addition, Guangdong province is one of the main acid rain distribution areas in China [41]; the lowest pH is 4.38 [42]. Studies have shown that pH is one of the important factors affecting the migration of heavy metals in soil [43,44,45]. Herein, chelator-induced phytoextraction for Pb- and Tl-contaminated soil remediation was investigated under simulated rainfall conditions (especially acid rain), in which *Zea mays* L. was used as the tolerant plants, and DTPA and oxalic acid were used as chelating agents. The leaching risk of Tl and Pb during the chelator-induced phytoextraction was highly concerned, providing important information on understanding the application of chelator-induced phytoextraction in Pb- and Tl-contaminated soil remediation. 

## 2. Materials and Methods

### 2.1. Collection and Preparation of Soil Samples

In this work, red soil samples were collected at depths of 0 to 20 cm from hillside land near a pyrite opencast mining site in Guangdong province (N 22°59’ 25.5”, E 112°00’ 40.5”), China. Samples were air dried. Large particles of rocks and plant residues were removed and sieved through a 4-mm nylon mesh after grinding. The physicochemical properties were analysed by the usual soil agrochemical method [46]. After the samples were digested in a 5:1 (*v/v*) mixture of HNO_3_ (guaranteed reagent, GR) and HF (GR) [31], the concentrations of Pb and Tl were measured by atomic absorption spectrometer (AAS) (iCE 3500; Fisher Scientific, Loughborough, UK). GBW070405 (GSS-5) was used as reference material for quality control in the determination of heavy metals in soils. The soil physicochemical properties are shown in Table 1.

To simulate soil contamination caused by mine wastewater, the soil samples were artificially spiked with solutions of Pb(NO_3_)_2_ and TlNO_3_, bringing the concentrations of Pb and Tl to 552.30 and 5.63 mg kg^−1^, respectively. Meanwhile, the soils were fertilized at 1.25 g pot^−1^ (N/P_2_O_5_/K_2_O = 15:15:15). Then, the spiked samples were placed in flowerpots (500 g pot^−1^) of the following dimensions: surface diameter of 13 cm, bottom diameter of 10 cm, and height of 10 cm. The soil in the pots was subjected to five cycles of wetting (70% water holding capacity) and drying (air drying), and was equilibrated for 15 days.

### 2.2. Glasshouse Experiment

The operation parameters of the glasshouse experiment are provided in Table S1. Three types of contaminated soil (Pb, Tl, Pb-Tl) were used in the experiment. Four conditions were set up for each contaminated soil: no plant- no chelating agent, plant- no chelating agent, plant- DTPA, and plant- oxalic acid. Three repetitions were set for each experimental condition. 

The seeds of maize “Zhengdan 958” were placed in a biochemical incubator at 25 °C for 3 days with a daily water supply. Germinated seeds with better growth characteristics were sown in the soil samples at depths of 2 to 3 cm in each experimental flower pot. The pots were then settled in a glasshouse with a stable temperature of 25 to 30 °C for 30 days. During cultivation, the water holding capacity was maintained at 70% by means of providing deionized water at 8:00 a.m. and 5:00 p.m. every day. After 30 days of growing, the chelating agents were added, and the added amount of DTPA and oxalic acid was 2.5 mmol kg^−1^. The leaching experiment was conducted on the third day when the activation effect of HMs was maximized according to previous experiments by Zhou [47].

*Z. mays* L. samples were harvested before the leaching experiment started. Before *Z. mays* L. was harvested, the chlorophyll content of the maize leaves was analysed using a plant nutrient measuring apparatus. Plant height, sixth leaf length, sixth leaf width, stem length, stem diameter, and leaf number were measured.

To acquire the dry weight of both the roots and the shoots, these parts of the plant were thoroughly washed and dried to a constant weight at 70 °C. Subsequently, samples were digested in the digestion system of HNO_3_ and H_2_O_2_, and were filtered through a 0.45-μm filter. The HMs were determined using an AAS. GBW 10012 (GSB-3) was used as reference material for quality control in the determination of heavy metals in plants.

### 2.3. Preparation of Acid Rain

A base acid rainwater was produced to simulate the acid rain according to the characteristics of acid rainwater studied by Zheng et al. [48]. (Table S2). Two artificial rainwater events were simulated with a mixture of H_2_SO_4_, HCl, and HNO_3_ in a molar ratio of SO_4_^2−^/Cl^−^/NO_3_^−^ of 6.1:1:0.7, by means of adjusting the base solution pH to either 4.5 or 6.5. The leaching scheme is shown in Table S3. 

### 2.4. Leaching Experiment

The influencing factors of the leaching experiment and designs included the type of heavy metal (Pb, Tl, and Pb-Tl), the plant (no plant and maize), the chelating agent (no chelating agents, DTPA, and oxalic acid), and the rainfall pH (pH 4.5 and 6.5, representing acid and non-acid rain, respectively). There were 24 treatments, and each treatment was repeated 3 times. Before each leaching experiment, the soil achieved 100% water holding capacity. Rainfall intensity was determined based on the Chinese heavy rain standards (i.e., 50 mm in 24 hours). According to the surface area in each pot, the total rainfall of a rainstorm was 600 ml, and the leachates were collected 5 times (120 ml/time) for the measurement of the pH and the amounts of HMs.

### 2.5. Data Processing and Statistical Methods

The BCF (bioconcentration factor) values, TF (translocation factor) values, and uptake amounts (amounts of HMs of shoots), were calculated to identify performance indicators for assessing the plant phytoextraction capacity. The calculation method of these indicators used the following formulas:

BCF = C_shoot_/C_soil_ (1)

Uptake = C_shoot_ × DW_shoot_ (2)

TF = C_shoot_/C_root_ (3)

where C_shoot_ is the concentration of Pb (or Tl) in plant dry aboveground biomass (mg kg^−1^, DW); C_soil_ is the concentration of Pb (or Tl) in the soil (mg kg^−1^, DW); DW_shoot_ is the dry aboveground biomass (g, DW); and C_root_ is the concentration of Pb (or Tl) in dry belowground biomass (mg kg^−1^, DW). 

Mapping was performed using the Origin software package (version 8.5; OriginLab, Northampton, MA, USA). Statistical and linear regression analyses were performed using GraphPad Prism version 6 (GraphPad Software, Inc., California, USA). Duncan’s multiple range test in SPSS 17 (SPSS Inc., Chicago, IL, USA) was used to compare the effects of different treatment schemes at a 0.05 significance level. Data were expressed as means plus orminus one standard deviation.

## 3. Results and Discussion

### 3.1. Effects of Chelating Agents on Plant Growth and Extraction of HMs

#### 3.1.1. Effects of HMs and Chelating Agents on the Growth of Maize

During the planting experiment, the growth status of corn was observed, heavy metal poisoning or chelating agent poisoning in the plant was not observed. The physiological indexes of corn are shown in Figure S1. The results were consistent with the results of Luo et al. [49]. It can be seen that no matter which chelating agent was added to the Pb-contaminated soil, the plant physiological indexes (including leaf number and leaf width, stem length and diameter, aboveground and underground dry weight and total dry weight) were significantly better than those in the Tl or Pb-Tl contaminated soils, suggesting the inhibitory effect of Tl on corn growth. In contrast, for the chlorophyll index, the Tl- and Pb-Tl contaminated soils were both better than the Pb-contaminated soil, which was consistent with the experimental results reported by Yao et al. [50]. who found that soil containing Tl (10 mg/kg) promoted photosynthesis in kale. Pu et al. [51] found that the content of chlorophyll was strongly affected by the oxidative stress tolerance, which is associated with Tl in the soil. In addition, compared with the control group of various types of contaminated soil, the physiological indexes of the plant were not changed significantly when DTPA or oxalic acid was added.

#### 3.1.2. Enrichment and Transfer of HMs by Chelators

The statistical results of Pb and Tl enrichment and transfer coefficients in single- and compound - contaminated soils are shown in Table 2 and Table 3. 

(i) In Pb-contaminated soil with the DTPA treatment, the BCF, uptake and TF coefficient of Pb-phytoextraction were significantly increased by 26%, 26%, and 45%, respectively, compared with the control group (Pb_Z-0_) without a chelating agent. The usage of oxalic acid increased the TF by 15%, while neither the BCF nor the uptake coefficient had significant increases. Synthetically, the type of chelators, especially DTPA, had a great influence on Pb-phytoextraction, which was similar to the results reported in previous works [24,52,53]. 

In Pb-Tl contaminated soil, compared with the results in the absence of chelators (Pb+Tl_Z-0_), the DTPA treatment remarkably increased the BCF, uptake, and TF coefficients of Pb by 47%, 143%, and 121%, respectively. The addition of oxalic acid showed similar rules, in which the BCF, uptake, and TF coefficients increased by 37%, 86%, and 100%, respectively.

Compared with the Pb-contaminated soil, the BCF, uptake, and TF of the Pb-Tl-contaminated soil were 51%, 85%, and 42% lower, respectively, without the addition of a chelating agent, and 43%, 71%, and 12% lower, respectively, with the addition of DTPA. When oxalic acid was added, the BCF and uptake coefficients of Pb-Tl-contaminated soil were 37% and 74% lower, respectively, and the TF remained almost unchanged. The results reflected that the Pb-extraction ability of plants was restrained due to the Tl from the Pb-Tl-contaminated soil.

(ii) In Tl-contaminated soil, adding DTPA greatly increased the BCF and uptake coefficients of Tl-phytoextraction by 17%, and 67%, respectively, compared with the control group (Tl_Z-0_) without a chelating agent, while this effect was not observed on the TF. The usage of oxalic acid had similar results, and the BCF and uptake coefficients increased by 11%, and 33%, respectively. Obviously, DTPA was much more effective than was oxalic acid, which was consistent with the results reported by Luo et al. [54,55].

In Pb-Tl-contaminated soil, compared to that without chelating agents (Pb+Tl_Z-0_), when DTPA was added, the BCF and uptake coefficients of Tl increased by 15% and 57%, respectively, while the TF significantly decreased by 14%. Similar results appeared when oxalic acid was added, where the TF declined by 16%, while the BCF slightly increased (not significant), and the uptake coefficients remarkably increased by 43%. The results inferred that chelators significantly enhanced Tl-phytoextraction, of which DTPA was better than oxalic acid.

When comparing Pb-Tl-contaminated soil with Tl-contaminated soil, the uptake coefficient of the former was 27% (DTPA) and 17% (oxalic acid) lower than that of the latter, which reflected that the Tl-extraction ability of plants was restrained due to the Pb from the Pb-Tl-contaminated soil. He et al. [56] found that the existence of different metals may inhibit absorption by plants. Potassium is a competitive inhibitor of Tl uptake, as they are both taken up by the same carrier system, which has a greater affinity for the carrier than for Tl [57]. 

### 3.2. Leaching Characteristics of Heavy Metals under Rainfall Conditions

#### 3.2.1. Effect of Acid Rain on the Total Amount of Leached Heavy Metals

**Figure 1:**
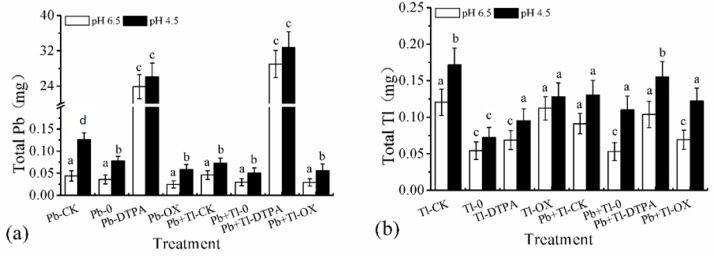
Cumulative Pb (**a**) and Tl (**b**) leached from soil by different treatments in leaching experiment. Error bars are the standard deviation (SD) (*n* = 3). Pb, Tl, and Pb+Tl indicate the types of heavy metal contaminations. CK = no maize; 0 = no chelating agents; DTPA = 2.5 mmol kg^−1^ soil; oxalic acid = 2.5 mmol kg^−1^ soil. Different superscript letters indicate a significant difference (*p* < 0.05).

The total leached amount of HMs by chelating is shown in Figure 1. For Pb single- contaminated soil and Pb-Tl compound—contaminated soil, except that the increase of Pb, leaching with DTPA was not significant under acid rainfall condition, the rest cases showed that the leached amount of Pb under acid rainfall condition was significantly greater than that under non-acid rainfall condition (about 2.07 times), indicating that the influence of acid rainfall was reduced after DTPA added. The results were similar with those of Lu et al. [32]. When being exposed to acid rainfall, the extracting fraction of HMs in soil was increased, leading to an increase in the total HMs in the leachate. For Tl single contaminated soil and Pb-Tl compound- contaminated soil, the change of Tl leaching under acid rainfall condition was more complex than that of Pb, but the leached amount under acid rainfall condition was greater than that under non-acid rain condition, and the average statistical result was 1.47 times of that under acid rainfall condition.

#### 3.2.2. Effect of Chelating Agent on the Total Amount of Leached Pb 

In Pb-contaminated soil, without the addition of chelating agents, the amount of Pb leached under non-planting conditions was higher than that with planting, as the extraction of Pb in maize reduced the amount of Pb in the leachate. Under planting conditions, the addition of DTPA increased the amount of Pb in the leachate by 338 and 672 times compared with that of no chelating agent treatment (corresponding to acid rain and non-acid rain, respectively), reaching 10% and 9% of the original amount of Pb in soil, which was also significantly higher than the results of non-planting conditions. This result showed that the addition of DTPA not only promoted the extraction by plants but also greatly increased the leakage of heavy metals. Lu et al. [31] found that the amount of Pb in the leachate with the EDTA treatment was 680 times that of the control sample, which agreed with the results in the present work. The addition of oxalic acid had no significant influence on the amount of leached Pb in the leachate. 

Similar phenomenon also appeared in the Pb-Tl-contaminated soil. In the Pb-Tl-contaminated soil with planting, the DTPA treatment increased the amount of Pb in the leachate by 659 and 978 times compared with that of the no chelating agents treatment (corresponding to acid rain and non-acid rain, respectively), reaching 13% and 12% of the original Pb amount in soil and accounting for 1.9 and 1.5 times that of the Pb-contaminated soil, as there could be competitive adsorption between Tl and Pb, which accelerates the leaching of some HMs [58]. The addition of oxalic acid had no significant effect on the leaching of Pb (Pb- or Pb-Tl-contaminated soil).

It can be concluded that the leaching of Pb in Pb single and compound contaminated soils increased significantly with the addition of DTPA, but the leaching of Pb in Pb single and compound contaminated soil with oxalic acid had no significant difference comparing with the control group without chelating agent under planting conditions. The results are presumably due to the higher stability constant of complexation of DTPA-Pb (lgK = 18.7) and the lower solubility of lead oxalate.

#### 3.2.3. Effect of Chelating Agent on the Total Amount of Leached Tl

For Tl-contaminated soil, the amount of heavy metal leaching in the condition of no planting and no chelating agent (Tl_-CK_) was higher than that in the condition of planting and added chelating agent (DTPA and oxalic acid). This result showed that the leakage of HMs could be controlled within the range of the amount of leakage before soil remediation when a chelating agent was added to promote the extraction by plants. After planting maize, the DTPA treatment increased the amount of Tl in the leachate by 31% and not significant (corresponding to the acid rain and non-acid rain, respectively) compared with the no chelating agent and planting conditions (Tl_-0_), reaching 4% and 3% of the original amount of Tl in the soil, respectively. Furthermore, the addition of oxalic acid increased the amount of Tl in the leachate by 78% and 106%, respectively, reaching 5% and 4% of the original amount of the Tl in soil.

For the Pb-Tl-contaminated soil without chelating agents, the amount of Tl leached under the non-planting condition was higher than that under the planting condition. Under the planting condition, the addition of DTPA increased the amount of Tl in the leachate by 42% and 96% (corresponding to acid rain and non-acid rain, respectively) compared with the no chelating agent treatment, reaching 6% and 4% of the original amount of the Tl in soil. The addition of oxalic acid had no significant influence on the amount of Tl in the leachate.

It can be concluded that compared with the control group without chelating agent under planting conditions, the leaching of Tl from Tl single and compound contaminated soils increased significantly in the presence of DTPA, and the leaching of Tl from Tl single contaminated soil increased significantly while it increased little from Tl compound contaminated soil in the presence of oxalic acid. The results may be ascribed to the fact that the combination of oxalic acid and Pb in contaminated soil consumed a lot of chelator.

#### 3.2.4. Change in the Leaching Process of Heavy Metals

**Figure 2:**
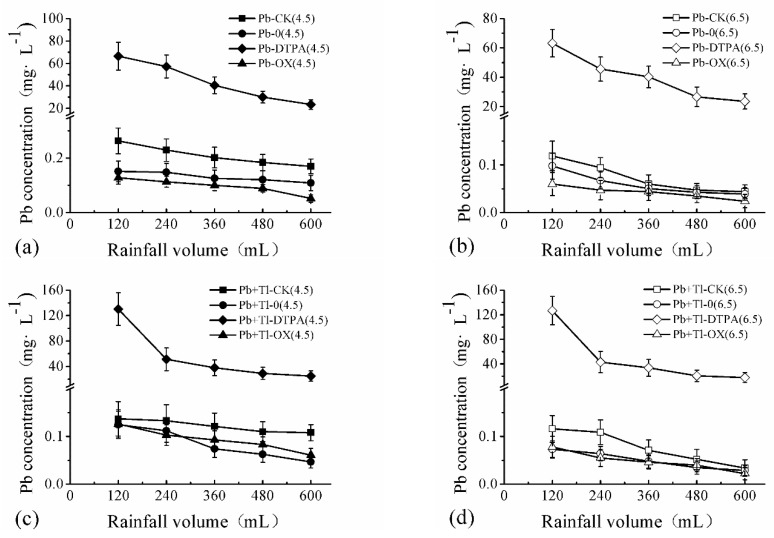

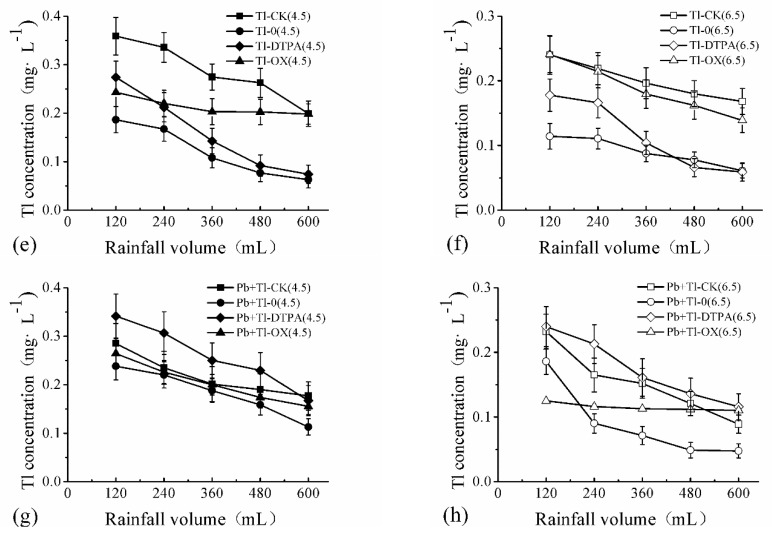
Concentrations of Pb (**a**–**d**) and Tl (**e**–**h**) in the leachates of DTPA- and oxalic acid-treated soil in the leaching experiment. Error bars are the standard deviation (SD) (*n* = 3). CK = no maize; 0 = no chelating agents; DTPA = 2.5 mmol kg^−1^ soil; oxalic acid = 2.5 mmol kg^−1^ soil.

Changes in the chelator-enhanced leaching of HMs are shown in Figure 2. The contents of HMs in the leachate showed a decreasing trend in accordance with the law of linear descent, which was consistent with the rule of HM leaching reported by Hu et al. [30]. It was mainly related to the linear increase of rainfall over time and the reduction of heavy metals in soil caused by continuous leaching. Most of the goodness-of-fit values were above 0.900, of which the linear regression equations are shown in Table S4.

#### 3.2.5. Effect of Chelating on Pb Concentration in the Leachate

In the Pb-contaminated soil without chelating agents, the concentration of Pb in the leachate with plants was lower than that without plants. This result was consistent with the results of Chen et al. [59], which was related to the absorption of plants. With the treatment of planting maize and the addition of DTPA, the average concentration and minimum concentration of Pb under acid rain and non-acid rain conditions were 43.91, 24.4, and 39.92, 23.3 mg/L, respectively. When adding oxalic acid, they were 0.092, 0.045 and 0.043, 0.024 mg/L, respectively.

In the Pb-Tl-contaminated soil without chelating agents, the results between the planting and no-planting conditions were similar with those of the Pb-contaminated soil. With the treatment of planting maize and the addition of DTPA, the average concentration and minimum concentration of Pb under acid rain and non-acid rain conditions were 55.3, 25.0 and 48.71, 19.20 mg/L, respectively. When adding oxalic acid, they were 0.093, 0.060 and 0.047, 0.020 mg/L, respectively. 

It can be found that with the adding oxalic acid, the average Pb concentration in the leached solution was very low, and is lower than the fourth-level standard of groundwater environmental quality in China (0.1 mg/L) (GB/T14848-2017) [60].

#### 3.2.6. Effect of Chelating on Tl Concentration in the Leachate

In the Tl-contaminated soil without chelating agents, the concentration of Tl in the leachate with plants was lower than that without plants. In the treatment of planting maize with the addition of DTPA, the average concentration and minimum concentration of Tl under acid rain and non-acid rain were 0.159, 0.075 and 0.117, 0.06 mg/L, respectively. With the addition of oxalic acid, they were 0.216, 0.198 and 0.187, 0.140 mg/L, respectively. Chen et al. [61] also found that EDDS had similar activation effects on Tl.

In Pb-Tl-contaminated soil without chelating agents, the observations were similar to those in the Tl-contaminated soil, regardless of whether planting occurred. Under the planting condition with the addition of DTPA, the average concentration and minimum concentration of Tl under acid rain and non-acid rain were 0.259, 0.165 and 0.171, 0.114 mg/L, respectively. With the addition of oxalic acid, they were 0.204, 0.156 and 0.113, 0.108 mg/L, respectively. 

It can be found that no matter whether DTPA or oxalic acid is added, after a heavy rain, the Tl content in the leakage water is still much higher than the limit of the Chinese Drinking Water Health Standards (GB5749-2006) (Tl 0.1 μg L^−1^) [62]. 

#### 3.2.7. Relation between Glasshouse and Leaching Experiments

According to the results from the greenhouse experiment and leaching experiment, it was found that there were two different relationships between plant uptake and leached amounts of HMs.

(i) For the Pb-contaminated soil with added DTPA, the uptake coefficient of Pb increased significantly by 26%, and the leached amount of Pb increased by 338 times (acid rain). There was a positive correlation between them. The Pb-Tl-contaminated soil with added DTPA had similar characteristics, i.e., the uptake coefficient significantly increased by 143%, and the leached amount of Pb increased by 659 times (acid rain). The results showed that DTPA not only promoted the absorption of heavy metals by plants, but also greatly enhanced the leakage of heavy metals under rainfall conditions, bringing risks to the groundwater environment. With the oxalic acid treatment in the Pb-contaminated soil, the uptake coefficient and Pb leaching amount did not change significantly, and for the Pb-Tl-contaminated soil, the uptake coefficient increased by 86%, and the leached amount of Pb decreased by 9% (not significant). The result indicated that a negative correlation existed between the plant extraction indexes and the leached amount. This relationship is beneficial for strengthening phytoextraction and preventing leakage risk.

(ii) For the Tl-contaminated soil in the presence of DTPA, the uptake coefficient of Tl was significantly increased by 67%, and the leached amount of Tl was increased by 31% (acid rain). For the Pb-Tl-contaminated soil, the uptake coefficient was significantly increased by 57%, and the leached amount of Tl was increased by 41%. There was a positive correlation between them. While improving the extraction efficiency of plants, the risk of HM leakage is also greatly increased. In the case of adding oxalic acid, for Tl-contaminated soil, the uptake coefficient increased significantly by 33%, and the leached amount of Tl increased by 78% (acid rain), and for the Pb-Tl-contaminated soil, the uptake coefficient increased by 43%, and the leached amount of Tl increased by 7% (not significant). There was also a positive correlation between HM extraction and leakage.

## 4. Conclusions 

Plant extraction efficiency and heavy metal leakage are related to chelating agent type, soil pollution type, and rainfall characteristics. Compared with the control group without chelating agent under planting conditions, the extraction efficiency of Pb, Tl increased by 86%, 43% from Pb-Tl -contaminated soil in the presence of oxalic acid, and there is no significant change in heavy metal leakage under rainfall conditions. It is the best phytoremediation scheme in this work, which is significant to improve the efficiency of soil remediation and to prevent environmental risks. Considering the other conditions, the extraction effect of chelating agent cannot match with the heavy metal leakage, or the extraction effect has not changed significantly, or the leakage of heavy metals was increased significantly, especially when DTPA was added to Pb-Tl- contaminated soil, the extraction efficiency of heavy metals increased by 143%, and the leached amount of Pb increased by 659 times, which has a great risk of groundwater pollution. In addition, under rainfall conditions, the HM concentration in the leachate showed a linear decreasing law. Acid rain promotes the leakage of HMs.

## Figures and Tables

**Table 1:** Soil physicochemical properties in the present study.

Physicochemical Properties	
pH_water_	4.5
pH_KCl_	4.2
Clay (%) <0.002 mm	16
Silt (%) 0.02–0.002 mm	33
Sand (%) >0.02 mm	51
Texture	Clay loam
Organic matter (g kg^−1^)	15.4
Cation exchange capacity (cmol kg^−1^)	3.3
Bulk density (g cm^−3^)	1.28
Total N (%)	0.08
Available N (mg kg^−1^)	37.8
Available P (mg kg^−1^)	0.9
Available K (mg kg^−1^)	12.1
Background total metal concentration (mg kg^−1^)	
Tl	0.63
Pb	52.30

**Table 2:** Enrichment and transfer coefficient of Pb in maize by chelator-enhanced remediation in glasshouse experiment.

Treatment	Shoot Concentrations (mg kg^−1^ Plant Tissue)	Root Concentrations (mg kg^−1^ Plant Tissue)	BCF *	Uptake (mg Shoot^−1^)	TF *
	Pb	Pb	Pb	Pb	Pb
Pb_Z-0_	192.66 ± 27.80 ^a^	587.85 ± 67.47 ^a^	0.39 ^a^	0.47 ^a^	0.33 ^a^
Pb_Z-DTPA_	243.92 ± 32.70 ^b^	504.87 ± 76.57 ^b^	0.49 ^b^	0.59 ^b^	0.48 ^b^
Pb_Z-OX_	204.69 ± 22.98 ^a^	539.88 ± 49.33 ^a^	0.41 ^a^	0.50 ^c^	0.38 ^c^
Pb+Tl_Z-0_	94.26 ± 12.27 ^c^	501.67 ± 40.59 ^b^	0.19 ^c^	0.07 ^d^	0.19 ^d^
Pb+Tl_Z-DTPA_	138.26 ± 22.15 ^d^	330.16 ± 44.91 ^c^	0.28 ^d^	0.17 ^e^	0.42 ^c^
Pb+Tl_Z-OX_	128.05 ± 17.04 ^d^	336.09 ± 16.40 ^c^	0.26 ^d^	0.13 ^f^	0.38 ^c^

Values represent means ± SD (*n* = 6); the different superscript letters (a, b, c, d, e, f) within a column indicate a significant difference at *p* < 0.05 according to Duncan’s multiple range test; CK = no maize; 0 = no chelating agents; DTPA = 2.5 mmol kg^−1^ soil; Oxalic acid = 2.5 mmol kg^−1^ soil; * no unit of measure.

**Table 3:** Enrichment and transfer coefficient of Tl in maize by chelator-enhanced remediation in glasshouse experiment.

Treatment	Shoot Concentrations (mg kg^−1^ Plant Tissue)	Root Concentrations (mg kg^−1^ Plant Tissue)	BCF *	Uptake (mg Shoot^−1^)	TF *
	Tl	Tl	Tl	Tl	Tl
Tl_Z-0_	94.00 ± 18.55 ^a^	372.46 ± 28.02 ^a^	18.80 ^a^	0.09 ^a^	0.25 ^a^
Tl_Z-DTPA_	109.75 ± 1.38 ^b^	433.45 ± 14.28 ^b^	21.95 ^b^	0.15 ^b^	0.25 ^a^
Tl_Z-OX_	104.72 ± 10.72 ^a^	430.81 ± 27.01 ^b^	20.95 ^b^	0.12 ^c^	0.24^a^
Pb+Tl_Z-0_	104.97 ± 13.41 ^a^	246.89 ± 31.32 ^c^	20.99 ^b^	0.07 ^d^	0.43 ^b^
Pb+Tl_Z-DTPA_	120.99 ± 11.61 ^c^	326.68 ±30.59 ^a^	24.20 ^c^	0.11 ^c^	0.37 ^c^
Pb+Tl_Z-OX_	108.23 ± 6.91 ^b^	299.48 ± 37.41 ^d^	21.65 ^b^	0.10 ^c^	0.36 ^c^

Values represent means ± SD (*n* = 6); the different superscript letters (a, b, c, d) within a column indicate a significant difference at *p* < 0.05 according to Duncan’s multiple range test. * no unit of measure.

## Supplementary Materials

The following are available online at https://www.mdpi.com/1660-4601/16/8/1328/s1, Figure S1: Effects of using DTPA (2.5 mmol L^−1^ soil) and oxalic acid (2.5 mmol L^−1^ soil) in different heavy metal contaminated soils on the morphological characteristics of maize, Table S1: Different treatments of the glasshouse experiment, Table S2: The chemical characteristics of rainfall at Yunfu, Guangdong Province, Table S3: Different treatments of the leaching experiment.
