# Supplementary material for: Insights into Heavy Metals Leakage in Chelator-Induced Phytoextraction of Pb- and Tl-Contaminated Soil
Source: Int J Environ Res Public Health. 2019 Apr 12;16(8):1328. doi: 10.3390/ijerph16081328 (PMC6518378; doi:10.3390/ijerph16081328)
Supplement: Supplementary file 1 [file ijerph-16-01328-s001.pdf]

International Journal of
Environmental Research and Public Health

MDPI

Supplementary Material

Insights into Heavy Metals Leakage in Chelator-Induced Phytoextraction of Pb- and Tl-Contaminated Soil

Xuexia Huang [1,2,3,4], Dinggui Luo [1,2,3,4,*], Xiangxin Chen [1], Lezhang Wei [1,2], Yu Liu [1,2], Qihang Wu [3,4], Tangfu Xiao [1], Xiaotao Mai [1], Guowei Liu [1] and Lirong Liu [1]

1 School of Environmental Science and Engineering, Guangzhou University, Guangzhou 510006, China; huangxuexia66@163.com (X.H.); chanxx0727@163.com (X.C.); wlz2016@gzhu.edu.cn (L.W.); liuyu@gzhu.edu.cn (Y.L.); tfxiao@gzhu.edu.cn (T.X.); maixiaotao@e.gzhu.edu.cn (X.M.); liuguowei@e.gzhu.edu.cn (G.L.); leonliutiming@e.gzhu.edu.cn (L.L.)
2 Linköping University—Guangzhou University Research Center on Urban Sustainable Development, Guangzhou University, Guangzhou 510006, China
3 Guangdong Provincial Key Laboratory of Radionuclides Pollution Control and Resources, Guangzhou University, Guangzhou 510006, China; wuqihang@gzhu.edu.cn
4 Key Laboratory for Water Quality and Conservation of the Pearl River Delta, Ministry of Education, Guangzhou University, Guangzhou 510006, China
* Correspondence: ldggq@gzhu.edu.cn

Table S1 Different treatments of the glasshouse experiment

No.	Treatment	HM	Plant	Chelator (mmol kg^{-1})
1	Pb_{ck}	Pb	None	0
2	Pb_0	Pb	Z. mays	0
3	Pb_{DTPA}	Pb	Z. mays	DTPA 2.5
4	Pb_{OX}	Pb	Z. mays	OX 2.5
5	Tl_{CK}	Tl	None	0
6	Tl_0	Tl	Z. mays	0
7	Tl_{DTPA}	Tl	Z. mays	DTPA 2.5
8	Tl_{OX}	Tl	Z. mays	OX 2.5
9	$Pb+Tl_{CK}$	Pb+Tl	None	0
10	$Pb+Tl_0$	Pb+Tl	Z. mays	0
11	$Pb+Tl_{DTPA}$	Pb+Tl	Z. mays	DTPA 2.5
12	$Pb+Tl_{OX}$	Pb+Tl	Z. mays	OX 2.5

Table S2 The chemical characteristics of rainfall at Yunfu, Guangdong Province

Component	Concentration (mg L^{-1})
NaCl	1.75
$(NH_4)_2SO_4$	2.24
KNO_3	2.14
$MgSO_4{\cdot}7H_2O$	5.79
$CaSO_4{\cdot}2H_2O$	24.48

Table S3 Different treatments of the leaching experiment

No.	Treatment	HM	Plant	Chelator (mmol kg^{-1})	Rainfall pH
1	$\mathrm{Pb_{ck}}$(4.5)	Pb	None	0	4.5
2	$\mathrm{Pb_{ck}}$(6.5)	Pb	None	0	6.5
3	$\mathrm{Pb_{0}}$(4.5)	Pb	Z. mays	0	4.5
4	$\mathrm{Pb_{0}}$(6.5)	Pb	Z. mays	0	6.5
5	$\mathrm{Pb_{DTPA}}$(4.5)	Pb	Z. mays	DTPA 2.5	4.5
6	$\mathrm{Pb_{DTPA}}$(6.5)	Pb	Z. mays	DTPA 2.5	6.5
7	$\mathrm{Pb_{OX}}$(4.5)	Pb	Z. mays	OX 2.5	4.5
8	$\mathrm{Pb_{OX}}$(6.5)	Pb	Z. mays	OX 2.5	6.5
9	$\mathrm{Tl_{CK}}$(4.5)	Tl	None	0	4.5
10	$\mathrm{Tl_{CK}}$(6.5)	Tl	None	0	6.5
11	$\mathrm{Tl_{0}}$(4.5)	Tl	Z. mays	0	4.5
12	$\mathrm{Tl_{0}}$(6.5)	Tl	Z. mays	0	6.5
13	$\mathrm{Tl_{DTPA}}$(4.5)	Tl	Z. mays	DTPA 2.5	4.5
14	$\mathrm{Tl_{DTPA}}$(6.5)	Tl	Z. mays	DTPA 2.5	6.5
15	$\mathrm{Tl_{OX}}$(4.5)	Tl	Z. mays	OX 2.5	4.5
16	$\mathrm{Tl_{OX}}$(6.5)	Tl	Z. mays	OX 2.5	6.5
17	$\mathrm{Pb{+}Tl_{CK}}$ (4.5)	Pb+Tl	None	0	4.5
18	$\mathrm{Pb{+}Tl_{CK}}$(6.5)	Pb+Tl	None	0	6.5
19	$\mathrm{Pb{+}Tl_{0}}$(4.5)	Pb+Tl	Z. mays	0	4.5
20	$\mathrm{Pb{+}Tl_{0}}$(6.5)	Pb+Tl	Z. mays	0	6.5
21	$\mathrm{Pb{+}Tl_{DTPA}}$(4.5)	Pb+Tl	Z. mays	DTPA 2.5	4.5
22	$\mathrm{Pb{+}Tl_{DTPA}}$(6.5)	Pb+Tl	Z. mays	DTPA 2.5	6.5
23	$\mathrm{Pb{+}Tl_{OX}}$(4.5)	Pb+Tl	Z. mays	OX 2.5	4.5
24	$\mathrm{Pb{+}Tl_{OX}}$(6.5)	Pb+Tl	Z. mays	OX 2.5	6.5

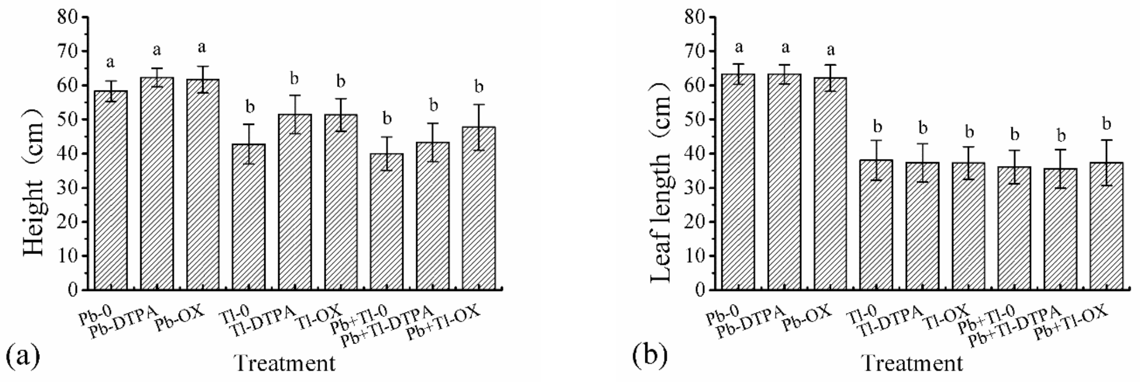

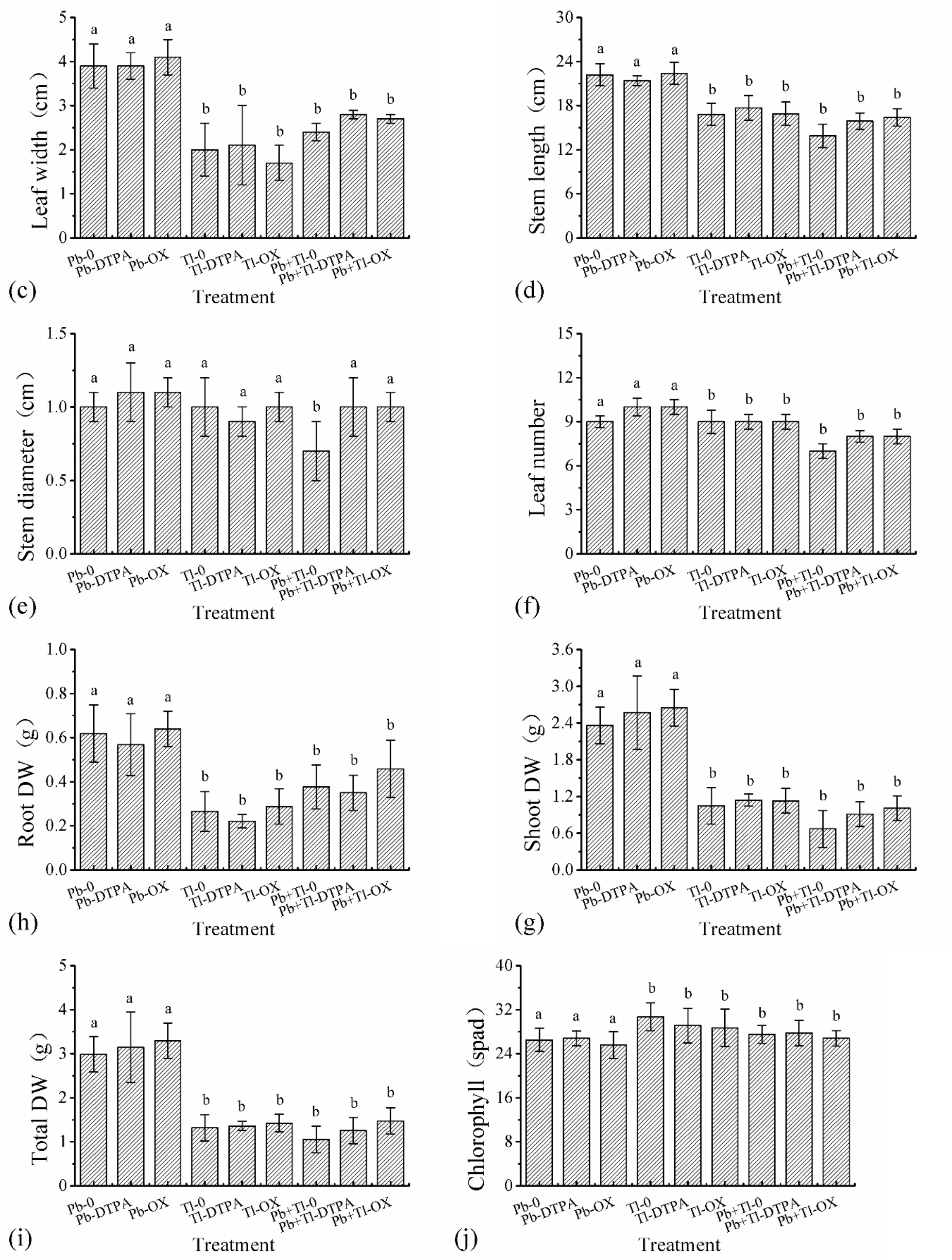

Fig. S1 Effects of using DTPA (2.5 mmol L^{-1} soil) and oxalic acid (2.5 mmol L^{-1} soil) in different heavy metal contaminated soils on the morphological characteristics of maize

Error bars are means ± SD (n=6)

(a) height, (b) leaf length, (c) leaf width, (d) stem length, (e) stem diameter, (f) leaf number, (g) shoot DW, (h) root DW, (i) total DW, (j) chlorophyll.

Letters on error bars in each plot indicate the difference compared with the blank treatment. The same letter

indicates that there was no significant difference ($P>0.05$), and different letters indicate a significant difference ($P<0.05$)

© 2019 by the authors. Submitted for possible open access publication under the terms and conditions of the Creative Commons Attribution (CC BY) license (http://creativecommons.org/licenses/by/4.0/).
